# Correlates of fentanyl preference among people who use drugs in Rhode Island

**DOI:** 10.1186/s12954-024-01089-5

**Published:** 2024-09-14

**Authors:** Siena C. Napoleon, Carolyn J. Park, Jacqueline Goldman, Yu Li, Jane A. Buxton, Alexandria Macmadu, Katie B. Biello, Julia Noguchi, Brandon D.L. Marshall

**Affiliations:** 1 Department of Behavioral and Social Health Sciences, Brown University School of Public Health, 121 South Main Street, Box G-S-121-3, Providence, RI 02912 USA; 2 Department of Epidemiology, Brown University School of Public Health, 121 South Main Street, Box G-S-121-2 Providence, Providence, RI 02912 USA; 3 School of Population and Public Health, University of British Columbia, 2206 East Mall, Vancouver, BC V6T 1Z3 Canada

**Keywords:** Fentanyl, Harm reduction, Public health, Opioid use disorder, Stimulant use disorder

## Abstract

**Background:**

Fentanyl is increasingly pervasive in the unregulated drug supply and is a driver of drug overdose deaths in the United States. The aims of this study were to characterize and identify correlates of fentanyl preference among people who use drugs (PWUD) in Rhode Island (RI).

**Methods:**

Using bivariate analysis, we examined associations between fentanyl preference and sociodemographic and psychosocial characteristics at baseline among participants enrolled in the RI Prescription Drug and Illicit Drug Study from August 2020-February 2023. Fentanyl preference was operationalized based on responses to a five-point Likert scale: “I prefer using fentanyl or drugs that have fentanyl in them.” Participants who responded that they “strongly disagree,” “disagree,” or were “neutral” with respect to this statement were classified as not preferring fentanyl, whereas participants who responded that they “agree” or “strongly agree” were classified as preferring fentanyl.

**Results:**

Among 506 PWUD eligible for inclusion in this analysis, 15% expressed a preference for fentanyl or drugs containing fentanyl as their drug of choice. In bivariate analyses, preference for fentanyl was positively associated with younger age, white race, lifetime history of overdose, history of injection drug use, past month enrollment in a substance use treatment program, past month treatment with medications for opioid use disorder, and preferences for heroin and crystal methamphetamine (all *p* < 0.05). Descriptive data yielded further insight into reasons for fentanyl preference, the predominant having to do with perceived effects of the drug and desire to avoid withdrawal symptoms.

**Conclusions:**

Only a relatively small subset of study participants preferred drugs containing fentanyl. Given the increased prevalence of fentanyl contamination across substances within the unregulated drug market, the result for PWUD is increasingly less agency with respect to choice of drug; for example, people may be forced to use fentanyl due to restricted supply and the need to mitigate withdrawal symptoms, or may be using fentanyl without intending to do so. Novel and more effective interventions for PWUD, including increased access to age-appropriate harm reduction programs such as fentanyl test strips and overdose prevention centers, are needed to mitigate fentanyl-related harms.

## Introduction

Fentanyl, a synthetic opioid that is estimated to be 50 to 100 times more potent than morphine, is approved by the United States Food and Drug Administration for clinical treatment of severe pain [1]. Due to the potency of fentanyl and other drug market factors, there is a notable presence of unregulated manufacture and consumption of this drug in the United States [2–4]. Illicitly manufactured fentanyl (IMF) produces analgesic and euphoric effects when consumed in small quantities and is sold through unregulated drug markets, often mixed with other opioid or stimulant drugs [5, 6]. 

Deaths involving IMFs are becoming increasingly prevalent. In the United States, more than 73,000 people died from overdoses involving synthetic opioids in 2022, a 25-fold increase from 2010 [1]. Rhode Island (RI) is one of the states hardest-hit by the opioid overdose crisis, where the incidence of fatal opioid overdose far exceeds the national average [7]. Fentanyl and fentanyl analogs were first detected in RI in 2013; [8] overdose deaths subsequently increased rapidly, with fentanyl contributing to over 78% of overdose deaths in 2023 [9]. Biosurveillance data of suspected overdose cases confirm that fentanyl and its analogs continue to be the main drivers of the opioid overdose epidemic in RI [10, 11]. In this context, public health efforts to promote fentanyl testing among people who use unregulated drugs in RI are ongoing [12, 13]. Trends related to opioid overdose mortality and specifically to fentanyl-involved overdose continue to evolve and many public health scientists are referring to the current landscape as the fourth wave of the opioid overdose epidemic, in which fentanyl is increasingly prevalent in stimulants such as cocaine and methamphetamine [14–18]. 

While some people who use drugs (PWUD) avoid fentanyl due to concerns about related harms [19–22], avoidance of fentanyl is becoming increasingly difficult in the context of the current unregulated drug market [23–25]. PWUD who are unaware that the products they consume contain fentanyl may be unprepared to manage side effects associated with fentanyl usage; most concerning among these side effects is resulting overdose that presents with rapid and severe onset of symptoms [26, 27]. Conversely, some PWUD may purposefully use fentanyl due to desired effects, financial resources, drug availability, decreased consumer autonomy, and physiological demand [28]. 

Limited prior research, both qualitative and quantitative, has found fentanyl preference to be associated primarily with PWUD who are younger in age [29–35], as well as the following variables: homelessness [29, 35], injection drug use [29, 32, 36], white race [30, 34, 35], diagnosis of severe mental illness [31], female sex [32], increased drug use frequency [34, 35], lifetime history of overdose [34, 35], recent stimulant use [34], and initiating opioid use with non-prescribed diverted medical opioids [35]. The association of fentanyl preference with these variables can be understood through a combination of sociocultural, economic, and biological factors; for example, PWUD frequently often develop tolerance to opioids, which necessitates the use of more potent opioids such as fentanyl to achieve the desired effect [28, 35, 36]. Similarly, a history of overdose is associated with high-risk pattern of drug use; [37, 38] individuals with these patterns of use may prefer fentanyl as their opioid tolerance increases. Finally, individuals who initiate opioid use with non-prescribed opioids, including diverted medical opioids, might be more likely to transition to fentanyl due to its availability on the unregulated market and its high potency [39, 40]. In this analysis, we seek to further explore sociodemographic, psychosocial, drug use, and behavioral correlates of fentanyl preference among a sample of PWUD in RI.

## Methods

### Study population

Baseline survey data were collected from participants in the RI Prescription Drug and Illicit Drug Study (RAPIDS) (NCT04372238) between August 2020 and February 2023.^37^ Inclusion criteria for participation in this research study were that participants must: be between 18 and 65 years of age; reside in RI; be able to complete interviews in English; be able to provide informed consent; and self-report past 30-day use of heroin, unregulated stimulants (e.g., powder cocaine, crack cocaine, methamphetamine), diverted or counterfeit prescription pills, or any drug by injection, regardless of treatment status. The RAPIDS study was approved by the Brown University Institutional Review Board. For this analysis, self-report data were extracted from the RAPIDS baseline survey. All analyses were performed using R Statistical Software (v4.2.3; R Core Team 2023) and the dplyr (v1.1.2; Wickham et al. 2023) package.

### Study variables

The primary outcome variable for this analysis was preference for fentanyl, operationalized based on responses to a five-point Likert scale: “I prefer using fentanyl or drugs that have fentanyl in them.” Participants who responded that they “strongly disagree,” “disagree,” or were “neutral” with respect to this statement were classified as not preferring fentanyl, whereas participants who responded that they “agree” or “strongly agree” were classified as preferring fentanyl. Next, participants who responded “Agree” or “Strongly Agree” to preferring were also asked, “Why do you prefer to use drugs with fentanyl in them?” and were read a list of answer choices (“Stronger or better high,” “Faster onset of high,” “Easier to prepare than other drugs,” “Cheaper than other opioids (including heroin and prescription pills),” “Dope sick or experiencing withdrawal symptoms,” “That’s all my dealer was selling,” “Curious about the effect of fentanyl,” “It is what I am used to using,” “Other”). If another reason for fentanyl preference was provided, this was recorded verbatim by the interviewer and recoded if appropriate for an existing category.

The independent variables for this analysis were selected based on review of prior literature and were as follows: chronological age, sex assigned at birth (male, female), current gender identity (cisgender, transgender or other gender), race (re-coded as White, non-White), ethnicity (Hispanic/Latine, not Hispanic/Latine), average monthly income (in USD; $0, <$1-500, $501–1500, >$1500), history of justice involvement (lifetime history of arrest, yes/no), and homelessness, defined as “not having a regular place to stay, and living in a shelter because of nowhere else to go, or living in a place not ordinarily used for sleeping, like an abandoned building, car, or park;” (ever homeless, homeless in past month).

Additionally, substance use history was assessed, including history of injection drug use (lifetime), history of overdose (lifetime/past month), recent engagement in drug or alcohol treatment (past month), use of medications for opioid use disorder (MOUD, defined as buprenorphine, Suboxone, or methadone; past month), and duration of overall substance use in years. Duration of overall substance use was operationalized based on responses to survey questions which asked separately about age of first use of benzodiazepines, prescription opioids, psychedelics, club drugs, crystal methamphetamine, powder cocaine, crack cocaine, heroin, MOUD, prescription stimulants, and other drugs. Participants were queried separately about whether substances had been administered via injection and at what age injection first occurred. For prescription medications, participants were asked about non-medical use, defined as use not directed by a physician. Responses indicating use of only alcohol, cannabis, e-cigarettes (i.e. Juul), or cough syrup were excluded from analysis. Earliest age of use of any substance, excluding alcohol, cannabis, e-cigarettes, or cough syrup, was used to calculate the onset of the overall duration of substance use. However, it is worth noting that some participants may have endorsed use of a substance, but did not indicate at which age it was first used, in which case there could be some misclassification (e.g., if someone reported use of heroin and cocaine, but refused to report the age at which they initiated cocaine). For the purposes of this analysis, we used the youngest age reported.

Participant preference of a given substance was based on self-report. If a participant endorsed lifetime use of a given substance, not including fentanyl, participants were then asked to select their single most preferred substance from among a list of all substances reported used; in other words, a participant could not report preference of a substance that a participant had not reported using in the past. For prescription medications, participants were queried about non-medical use only.

Participants’ overdose history was operationalized as a single variable with three levels: (1) no lifetime history of overdose, (2) lifetime history of overdose, but no past month overdose, and (3) past month overdose. For the purposes of reporting about participants’ frequency of use, regular use of a substance was defined as being equal to four or more days of injection or non-injection use of a given substance in the past month. For prescription medications, participants were queried about non-medical use only.

Finally, history of mental health or cognitive disorder (lifetime) was collected. For the mental health history portion of the survey, participants were able to endorse having had multiple diagnoses.

### Data analysis

Among participants who preferred fentanyl, descriptive statistics regarding reason are reported. Each variable was tested for significant association with fentanyl preference using a chi squared test (or Fisher’s Exact test for groups with *n* < 10) (α = 0.05). For continuous variables such as age, a student’s T-test was performed.

## Results

There were 506 eligible participants in the study sample. The mean age of participants in the total study sample was 43.3 years (SD = 11.3); 67% identified as male gender, and 55% as white. Study cohort characteristics and demographic data, including data on homelessness, average monthly income, and mental health are reported in Table 1; participants are grouped in Table 1 according to fentanyl preference. As shown, only about 15% of the study sample (i.e., 76 participants) endorsed a preference for fentanyl.

**Table 1:** Sociodemographic and other characteristics associated with preferring fentanyl among 506 people who use drugs in Rhode Island

Characteristic	Total (%) (*n* = 506)	Preferred fentanyl		*p* - value
		Yes (%) (*n* = 76)	No (%) (*n* = 430)	
Sociodemographics*				
Age in years (mean, SD)	43.3 (11.3)	38.9 (9.5)	44.1 (11.3)	< 0.001
Male sex assigned at birth	340 (67.2)	49 (64.5)	291 (67.7)	0.678
Cisgender	485 (96.2)	74 (97.4)	411 (95.6)	0.334
White race	278 (54.9)	55 (72.4)	223 (51.9)	0.002
Not Hispanic/Latine	105 (20.8)	10 (13.2)	95 (22.1)	0.104
Ever homeless	474 (93.7)	72 (94.7)	402 (77.7)	0.804
Currently homeless	261 (51.8)	45 (59.2)	216 (50.2)	0.200
**Substance use**				
History of IVDU (lifetime)	277 (54.7)	59 (77.6)	218 (50.7)	< 0.001
History of overdose (lifetime)	228 (45.7)	21 (27.6)	207 (48.1)	< 0.001
Duration of use in years (mean, SD)	26.6 (11.9)	23.1 (9.7)	27.2 (12.1)	0.004
Currently in treatment program	142 (28.2)	29 (38.2)	113 (26.3)	0.050
Currently treated with MOUD	125 (24.7)	29 (38.2)	96 (22.3)	0.005
**Mental Health** ^†^				
Never diagnosed with mental health or cognitive disorder	138 (27.3)	24 (31.6)	114 (26.5)	0.405
Obsessive-compulsive disorder	42 (8.3)	5 (6.6)	37 (8.6)	0.658
Eating disorders (anorexia, bulimia)	14 (2.8)	2 (2.6)	12 (2.8)	1.000
Depressive disorder	220 (43.5)	32 (42.1)	188 (43.7)	0.892
Bipolar disorder	176 (34.8)	21 (27.6)	155 (36.1)	0.197
Psychosis	75 (14.8)	11 (14.5)	64 (14.9)	1.000
Anxiety disorders	220 (43.5)	36 (47.4)	184 (42.8)	0.538
ADD/ADHD	115 (22.7)	15 (13.0)	100 (23.3)	0.599

* “Don’t know/refuse to answer” responses excluded

† Respondents were able to select > 1 diagnosis

Mean age differed significantly at the α = 0.05 level between participants who endorsed fentanyl preference (38.9 years, SD = 9.5) and those who did not (44.1 years, SD = 11.3, *p* < 0.001). Race also differed significantly between participants who endorsed preference of fentanyl and those who did not (*p* = 0.002). Specifically, of those who reported preferring fentanyl, 72.4% identified as white, compared to 51.9% of participants who did not prefer fentanyl.

Overdose history differed significantly between participants who endorsed preference of fentanyl and those who did not, with 72.4% of those who preferred fentanyl experiencing lifetime history of overdose compared to 50.1% of those who did not prefer fentanyl (*p* < 0.001). Similarly, participants who endorsed preference for fentanyl were more likely to have lifetime injection drug use (*p* < 0.001) and have a shorter history of substance use compared to those who did not prefer fentanyl (23.1 years, SD = 9.7 vs. 27.2 years, SD = 12.1, respectively; *p* = 0.004).

Approximately 27.3% of all study participants reported having never been diagnosed with a mental health or cognitive disorder. Of these, 31.6% of participants who preferred fentanyl reported having never been diagnosed with a mental health or cognitive disorder and 26.5% of participants who did not prefer fentanyl reported having never been diagnosed with a mental health or cognitive disorder; these differences were not statistically significant.

Participants who endorsed a preference for fentanyl were more likely to be currently utilizing drug or alcohol treatment (*p* = 0.050) and MOUD (*p* = 0.005).

Participants’ substance use frequency and preferences were assessed and are reported in Table 2. Participants who preferred fentanyl were more likely to regularly use non-medical benzodiazepines (*p* < 0.001), crystal methamphetamine (*p* < 0.001), heroin (*p* < 0.001), non-medical MOUD (*p* = 0.033), psychedelics (*p* = 0.033), powder cocaine (*p* = 0.002), non-medical prescription opioids (*p* < 0.001), and non-medical prescription stimulants (*p* = 0.039).

**Table 2:** Frequency and preference of substance use behaviors among 506 people who use drugs in Rhode Island

Characteristic	Total (%) (*n* = 506)	Preferred fentanyl		*p* - value
		Yes (%) (*n* = 76)	No (%) (*n* = 430)	
Regular use endorsed*				
Benzodiazepines^†^	111 (21.9)	31 (40.8)	80 (18.6)	< 0.001
Crack cocaine	308 (60.9)	49 (64.5)	259 (60.2)	0.568
Club drugs	16 (3.2)	5 (6.6)	11 (2.6)	0.076
Crystal methamphetamine	82 (16.2)	23 (30.3)	59 (13.7)	< 0.001
Heroin	161 (31.8)	54 (74.1)	107 (24.9)	< 0.001
MOUD^†^	65 (12.9)	16 (21.1)	49 (11.4)	0.033
Psychedelics	9 (1.8)	4 (5.3)	5 (1.2)	0.033
Powder cocaine	136 (26.9)	32 (42.1)	104 (24.2)	0.002
Prescription opioids^†^	124 (24.5)	34 (44.7)	90 (20.9)	< 0.001
Prescription stimulants^†^	66 (13.0)	16 (21.1)	50 (11.6)	0.039
**Preference endorsed**				
Benzodiazepines^†^	26 (5.1)	6 (7.9)	20 (4.7)	0.798
Crack cocaine	177 (35.0)	19 (25.0)	158 (36.7)	0.081
Club drugs	1 (0.2)	0 (0.0)	1 (0.2)	1.000
Crystal methamphetamine	35 (6.9)	3 (4.0)	32 (7.4)	0.020
Heroin	122 (24.1)	49 (64.5)	73 (17.0)	< 0.001
MOUD^†^	8 (1.6)	2 (2.6)	6 (1.4)	1.000
Psychedelics	1 (0.2)	0 (0.0)	1 (0.2)	1.000
Powder cocaine	44 (8.7)	6 (7.9)	38 (8.8)	0.531
Prescription opioids^†^	16 (3.2)	6 (7.9)	10 (2.3)	0.118
Prescription stimulants^†^	3 (0.6)	1 (1.3)	2 (0.5)	0.467

* Regular use defined as total use > = 4 days of injection and non-injection use

† Denotes non-medical use

Reasons for fentanyl preference among participants who preferred fentanyl (*n* = 76) are reported in Table 3. The most frequently endorsed reason for preferring fentanyl was “Stronger or faster high” (80.3%), followed by “Faster onset of high” (40.8%). Other reasons given for fentanyl preference by participants included: “If I use just heroin I still have withdrawals,” “Dope is too weak,” “[Fentanyl is] better for pain relief with less of a high,” “Husband uses fentanyl,” and “[It’s] better to use drugs with fentanyl in them than ‘straight fentanyl’” (all other reasons endorsed at a rate of 1.3%).

**Table 3:** Reasons endorsed for fentanyl preference among 506 people who use drugs in Rhode Island

Characteristic	***n***	%^†^
Reason*		
Stronger or better high	61	80.3
Faster onset of high	31	40.8
Dope sick or experiencing withdrawal symptoms	26	34.2
It is what I am used to using	24	31.6
Cheaper than other opioids	18	23.7
That’s all my dealer was selling	13	17.1
Easier to prepare than other drugs	5	6.6
Other	5	6.6
Curious about the effect of fentanyl	4	5.3

* Participants were able to select > 1 reason

† Percentage is of 76 participants who selected “agree” or “strongly agree” with the statement “I prefer fentanyl”

## Discussion

This analysis found that PWUD in RI who endorsed a preference for fentanyl were more likely to be younger in age, be of white race, have a lifetime history of overdose, have a lifetime history of injection drug use, have shorter overall lifetime substance use career duration, attend a drug or alcohol treatment program, and have prior experience with MOUD. Participants who endorsed a preference for fentanyl were also more likely to report regular use of other substances (e.g., powder cocaine), and were more likely to endorse a preference for crystal methamphetamine and heroin.

Behaviorally, we found that preference for fentanyl appears to be derived from users’ perceptions to the perceived positive effects of the drug: participants who did express a preference for fentanyl largely cited both “stronger or better high” and “faster onset of high” as top reasons cited for endorsing fentanyl preference. On the other hand, another common reason cited for fentanyl preference was related to the avoidance of negative effects of drug usage, as participants who preferred fentanyl additionally cited “dope sick or experiencing withdrawal symptoms” as a reason for this preference. For PWUD, fentanyl may thus be effectively unavoidable for two reasons: its pervasiveness in the unregulated drug supply and its efficacy at relieving withdrawal symptoms in cases of dependence. Overall, these findings align with previously-published literature on fentanyl preference.

While it is possible that a growing preference for fentanyl and its novel analogues have led to increased demand for this product within the unregulated drug market, it is also possible that increased supply of fentanyl created demand by restricting the options for many PWUD. The analyses presented in this study show indications that both explanations may be true, although these analyses are limited insofar as being able to demonstrate a causal link in favor of one explanation over the other. These findings support the findings of related studies in the literature insofar as revealing a statistical association between fentanyl preference and younger age among users [29–35], increased drug use frequency [34, 35], injection drug use [29, 32, 36], white race [30, 34, 35], lifetime history of overdose [34, 35], and recent stimulant use [34]. 

Only 15% of our sample preferred fentanyl, suggesting that a relatively small subpopulation of PWUD exists within the RI area that prefers fentanyl. Given the amount of fentanyl that is available within the drug market currently, it is thus surprising that the demand for this product is relatively low. These findings also suggest that this subpopulation prefers fentanyl due to its increased potency and the need for relieving withdrawal symptoms. That this subpopulation is statistically younger and has, on average, a shorter overall substance use career duration suggests that preference for fentanyl may also be driven by lack of agency over substance used. We draw this conclusion given that these younger individuals had a relatively shorter career in which less potent opioids were used prior to fentanyl becoming prevalent in the unregulated drug market. As such, evidence-based interventions prioritizing younger PWUD are warranted. These may include increased distribution of fentanyl test kits and education regarding overdose prevention and response strategies.

Although preference for fentanyl may be limited to a small subpopulation, the distribution of fentanyl within the drug supply in RI is nonetheless widespread [41, 42], making this substance difficult for PWUD and do not prefer fentanyl to avoid it. It is critical that all PWUD be offered the opportunities, resources, and spaces to engage in harm reduction and overdose prevention strategies, including using fentanyl test strips, not using alone, accessing a safe supply, carrying naloxone, and being prepared to administer lifesaving measures (such as CPR and rescue breaths) in the event of an overdose.

Public health institutions and partners play a critical role in ensuring that PWUD have access to harm reduction education and supplies. Regardless of preference, all PWUD are currently at risk of accidental fentanyl overdose due to the widespread distribution of this substance and increased frequency of contamination with other opioids and stimulants. As RI is poised to be the first state in the nation to open a state-sanctioned overdose prevention center [43], the increasing risk of fentanyl overdose underlines that fact that harm reduction services are urgently needed and are deserving of both political support and funding in order to save the lives of Rhode Islanders.

This study should be interpreted with several limitations in mind. As previously mentioned, the analytic methods used in study render it unable to provide a causal explanation for fentanyl preference. Secondly, white, male, non-Hispanic, older adult (aged 35 + years) enrollees were statistically overrepresented in the study cohort. The study’s findings may thus be limited in generalizability due to the lack of diversity in its participant demographics, and this demographic homogeneity raises concerns about the applicability of the results to more diverse populations. Finally, this study was completed with a participant cohort of PWUD within a limited geographic area within the Northeastern region of the United States, and so the findings may not be applicable to other locations.

Despite the limitations, while only a small proportion of PWUD in RI explicitly prefer fentanyl, its pervasive presence in the drug market and its effects make it a significant factor for many users. The findings underscore the need for targeted harm reduction strategies, particularly for younger individuals and those with shorter substance use careers, to mitigate the risks associated with fentanyl use. Public health efforts should focus on expanding access to harm reduction resources, such as fentanyl test strips and naloxone, to address the widespread risk of accidental overdose and support the broader community of PWUD.

## Data Availability

No datasets were generated or analysed during the current study.

## Abbreviations

IMF: Illicitly manufactured fentanyl
RI: Rhode Island
PWUD: People who use drugs
RAPIDS: Rhode Island Prescription Drug and Illicit Drug Study
MOUD: Medications for opioid use disorder
CI: Confidence interval
SD: Standard deviation
